# Aberrant PLN-R14del Protein Interactions Intensify SERCA2a Inhibition, Driving Impaired Ca^2+^ Handling and Arrhythmogenesis

**DOI:** 10.3390/ijms23136947

**Published:** 2022-06-22

**Authors:** Elizabeth Vafiadaki, Kobra Haghighi, Demetrios A. Arvanitis, Evangelia G. Kranias, Despina Sanoudou

**Affiliations:** 1 Molecular Biology Division, Biomedical Research Foundation of the Academy of Athens, 11527 Athens, Greece; lvafiadaki@bioacademy.gr (E.V.); arvanitd@bioacademy.gr (D.A.A.); kraniaeg@ucmail.uc.edu (E.G.K.); 2 Department of Pharmacology and Systems Physiology, University of Cincinnati College of Medicine, Cincinnati, OH 45267-0575, USA; haghigk@ucmail.uc.edu; 3 Clinical Genomics and Pharmacogenomics Unit, 4th Department of Internal Medicine, Attikon Hospital Medical School, National and Kapodistrian University of Athens, 11527 Athens, Greece; 4 Center for New Biotechnologies and Precision Medicine, Medical School, National and Kapodistrian University of Athens, 11517 Athens, Greece

**Keywords:** phospholamban, R14del mutant, cardiomyopathy, cardiac arrhythmias

## Abstract

Phospholamban (PLN), a key modulator of Ca^2+^-homeostasis, inhibits sarcoplasmic reticulum (SR) calcium-ATPase (SERCA2a) and regulates cardiac contractility. The human *PLN* mutation R14del has been identified in arrhythmogenic cardiomyopathy patients worldwide and is currently extensively investigated. In search of the molecular mechanisms mediating the pathological phenotype, we examined PLN-R14del associations to known PLN-interacting partners. We determined that PLN-R14del interactions to key Ca^2+^-handling proteins SERCA2a and HS-1-associated protein X-1 (HAX-1) were enhanced, indicating the super-inhibition of SERCA2a’s Ca^2+^-affinity. Additionally, histidine-rich calcium binding protein (HRC) binding to SERCA2a was increased, suggesting the inhibition of SERCA2a maximal velocity. As phosphorylation relieves the inhibitory effect of PLN on SERCA2a activity, we examined the impact of phosphorylation on the PLN-R14del/SERCA2a interaction. Contrary to PLN-WT, phosphorylation did not affect PLN-R14del binding to SERCA2a, due to a lack of Ser-16 phosphorylation in PLN-R14del. No changes were observed in the subcellular distribution of PLN-R14del or its co-localization to SERCA2a. However, in silico predictions suggest structural perturbations in PLN-R14del that could impact its binding and function. Our findings reveal for the first time that by increased binding to SERCA2a and HAX-1, PLN-R14del acts as an enhanced inhibitor of SERCA2a, causing a cascade of molecular events contributing to impaired Ca^2+^-homeostasis and arrhythmogenesis. Relieving SERCA2a super-inhibition could offer a promising therapeutic approach for PLN-R14del patients.

## 1. Introduction

Phospholamban (PLN) is a 52-amino-acid transmembrane protein that inhibits the sarcoplasmic reticulum (SR) calcium ATPase (SERCA2a) and, thus, regulates SR Ca^2+^ cycling and cardiac contractility [1]. To date, a number of human mutations have been reported in the *PLN* gene [2]; however, the heterozygous deletion of arginine at amino acid residue 14 (R14del) has recently attracted considerable attention [3,4]. This variant was originally identified in a large Greek family with dilated cardiomyopathy (DCM) and heart failure and is associated with prominent arrhythmias and premature death [5]. Since then, the *PLN* mutationR14del has been identified in an increasing number of patients around the world [6,7,8,9], with this *PLN* genetic variant being the most prevalent cardiomyopathy-related mutation in the Netherlands (>1000 patients) [4,9,10,11].

At the molecular level, the pathophysiological mechanisms underlying PLN-R14del remain largely unclear. Based on initial findings from PLN-R14del overexpression in a heterologous cell culture system as well as in the mouse heart, PLN-R14del was proposed to exert super-inhibitory effects on SERCA2a activity, leading to cardiac remodeling and early death [5]. Additional evidence has recently emerged from numerous studies on human patients and/or patient-derived cardiomyocytes (iPSC-CMs), and various PLN-R14del animal models [12,13,14,15,16,17,18]. These studies have revealed multiple defects associated with the PLN-R14del mutation, including impaired Ca^2+^ homeostasis [12,13,14,16,18], electrical remodeling [19], unfolded protein response (UPR) activation [20], and protein aggregation [15,21,22].

**Figure 1:**
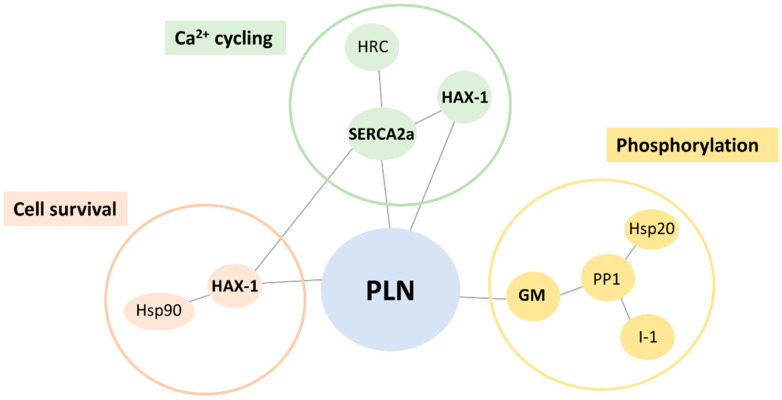
Schematic representation of key PLN associations. PLN interacts directly with SERCA2a, HAX1, and GM (shown in bold). These proteins are associated with additional binding partners that can be grouped into distinct clusters exerting different physiological functions.

PLN is known to exert its inhibitory function on SERCA2a through direct interactions at both its cytosolic and transmembrane regions [1]. Detailed alanine-scanning mutagenesis and structural studies have pinpointed specific amino acid residues involved in this association [21,22,23]. While PLN was originally believed to solely bind to SERCA2a, over the last decade, additional PLN-binding partners have been identified, including HS-1-associated protein X-1 (HAX-1) and GM (also known as RGL or PPP1R3) [24,25]. Both proteins have been demonstrated to bind directly to PLN, modulating its activity either by enhancing its inhibitory action on SERCA2a [25,26] or by regulating its phosphorylation status [24]. As SERCA2a and GM are, in turn, associated with additional binding partners that mediate different physiological functions, it becomes apparent that PLN is directly or indirectly associated with a complex multimeric network of proteins (Figure 1).

In the current study, we focused on the tentative first steps of the pathogenetic process, by examining the impact of the R14del mutation on known PLN protein interactions. Our findings reveal alterations in PLN-R14del associations to known PLN-interacting partners, with direct implications on key functional aspects such as Ca^2+^ handling. These aberrations may underlie the pathological mechanisms contributing to impaired Ca^2+^ homeostasis and arrhythmogenesis in PLN-R14del hearts.

## 2. Results

### 2.1. Altered PLN-R14del Interactions to Known PLN-Binding Partners

**Figure 2:**
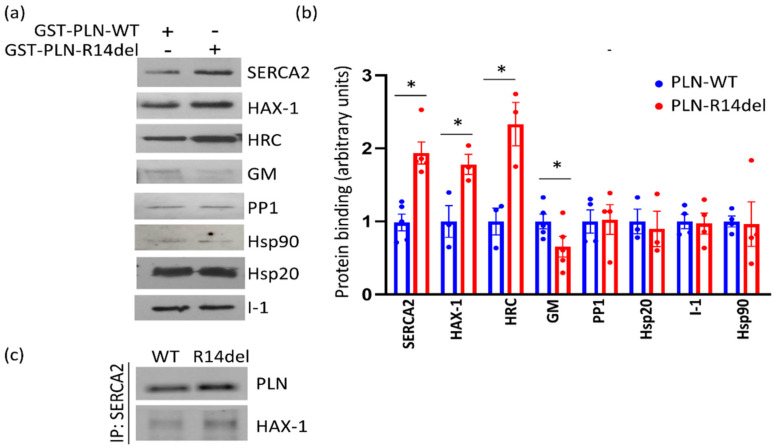
PLN-R14del exhibits altered interactions to known PLN-binding partners. (**a**) Pulldown assays using PLN-WT or PLN-R14del recombinant proteins and mouse cardiac extracts determined enhanced PLN-R14del binding to SERCA2a, HAX-1, and HRC and reduced binding to GM. (**b**) Quantification of protein interactions detected by pull-down assays (*n* = 4; * *p* < 0.05 vs. PLN-WT; *t* test, two-tailed). (**c**) Immunoprecipitation assays were performed in cardiac extracts from WT or the humanized PLN-R14del mouse model, confirming an increased (~1.5 fold) association of PLN-R14del to SERCA2a and HAX-1 (*n* = 3 pool of hearts from WT or PLN-R14del mice; experiments performed in duplicate).

In order to explore the molecular defects instigated by the PLN-R14del mutation, we examined for potential alterations in PLN-R14del association to its key direct interacting partners (Figure 1). Toward this, we performed pull-down assays using GST-PLN-WT or GST-PLN-R14del recombinant proteins and wild-type mouse cardiac extracts. Western blot analysis determined a reduced binding of PLN-R14del to the phosphatase targeting subunit GM, while SERCA2a and HAX-1 interactions were found to be enhanced (Figure 2a,b). These alterations were confirmed by immunoprecipitation assays in the humanized PLN-R14del mouse model hearts (Figure 2c). These findings suggest that through its increased binding to HAX-1 and SERCA2a, PLN-R14del may cause stronger inhibition on the Ca^2+^-affinity of SERCA2a for Ca^2+^, which would result in suppressed SERCA2a activity and decreased SR Ca^2+^ uptake.

SERCA2a, HAX-1, and GM are known to bind to several other proteins, with distinct physiological functions (Figure 1). We, therefore, expanded our study to assess for potential aberrations amongst the most immediate indirect associations of PLN-R14del. Toward this, we focused our investigations to particular proteins, which we previously determined to significantly impact cardiac function [24,25,26,27,28,29,30]. Among these, only the histidine-rich calcium binding protein (HRC) association was found to be altered, showing increased binding in the presence of PLN-R14del, which indicates increased inhibition of SERCA2a’s maximal rate (Vmax) [31] (Figure 2a,b). Overall, these findings reveal aberrations in PLN-R14del interactions to multiple key Ca^2+^ cycling-related proteins that will impact on SERCA2a function by suppressing its activity and will, therefore, lead to impaired SR Ca^2+^ cycling and depressed cardiac function.

### 2.2. PLN-R14del Association to SERCA2a Remains Enhanced upon Phosphorylation

**Figure 3:**
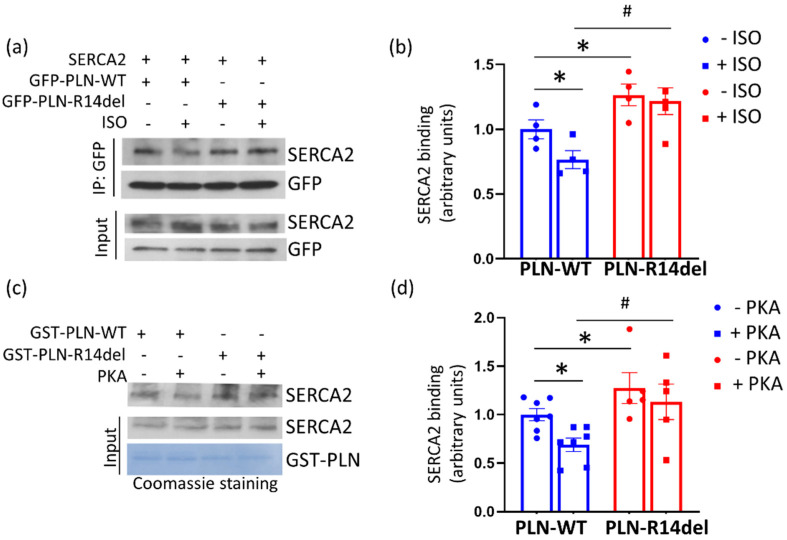
PLN-R14del association to SERCA2a remains unaltered upon phosphorylation. (**a**) Immunoprecipitation assays in transfected HEK 293 cells determined that ISO treatment resulted in the diminished interaction of PLN-WT with SERCA2a, while the interaction of PLN-R14del with SERCA2a was not altered. (**b**) Quantification of SERCA2a binding (*n* = 4; * *p* < 0.05 vs. PLN-WT without ISO; # *p* < 0.05 vs. PLN-WT with ISO; *t* test, two-tailed). (**c**) Similar findings were also detected by pull-down assays in mouse cardiac extracts that were phosphorylated by PKA treatment. (**d**) Quantification of SERCA2a binding (*n* = 5; * *p* < 0.05 vs. PLN-WT without PKA; # *p* < 0.05 vs. PLN-WT with PKA; *t* test, two-tailed).

Protein phosphorylation is known to relieve the inhibitory effect of PLN on SERCA2a activity [1]. Given the enhanced binding of PLN-R14del to SERCA2a, we next examined the impact of phosphorylation on the PLN-R14del/SERCA2a interaction. This was assessed in HEK 293 cells that had been co-transfected with GFP-PLN-WT and SERCA2a or GFP-PLN-R14del and SERCA2a plasmids. Protein phosphorylation at Ser-16 was performed by isoproterenol (ISO) treatment and its effect on the PLN-R14del/SERCA2a interaction was determined by immunoprecipitation assays. Western blot analysis revealed that the ISO treatment of PLN-WT resulted in reduced binding to SERCA2a, in accordance with previous reports [32,33,34]. In contrast, the phosphorylation of PLN-R14del by ISO treatment did not appear to affect its interaction with SERCA2a, which remained increased and at similar levels to the nonphosphorylated sample (Figure 3a,b). Similar findings were also obtained by pull-down assays in wild-type mouse cardiac extracts that had been phosphorylated by PKA treatment at the Ser-16 site (Figure 3c,d). Collectively, these results indicate that PLN-R14del binding to SERCA2a is not regulated by phosphorylation, therefore suggesting that it acts as a nonreversible inhibitor of SERCA2a that constitutively suppresses its activity.

### 2.3. PLN-R14del phosphorylation at Residue Ser-16 Is not Attained, due to Disruption of the PKA Motif

**Figure 4:**
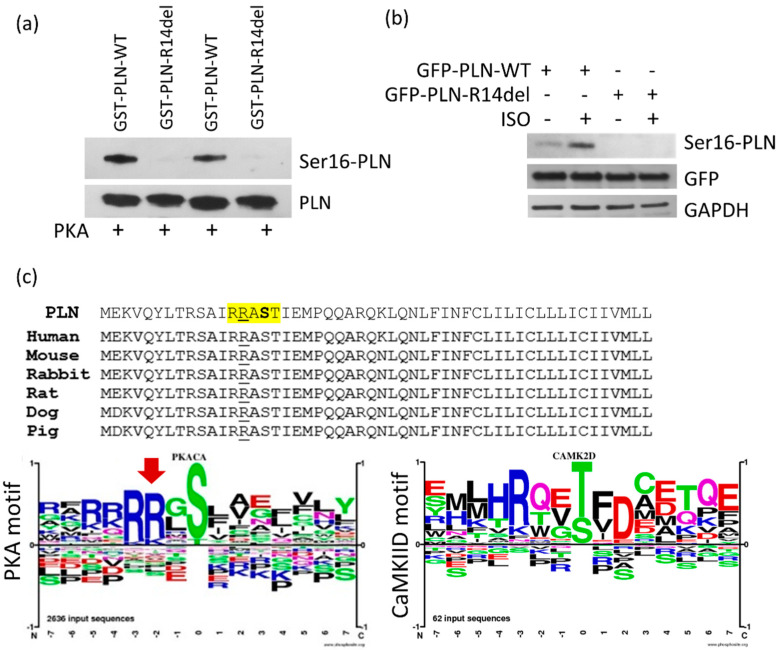
PLN-R14del is not phosphorylated at residue Ser-16. (**a**) Lack of in vitro phosphorylation of PLN-R14del by PKA as determined by the Ser16-phosphorylated PLN antibody. (**b**) Similarly, no phosphorylation was detected in transfected HEK 293 cells after ISO treatment. (**c**) Human PLN sequence (top) and alignment with proteins from different species. Highlighted residues surround the phosphorylation site at Ser-16 (shown in bold), while amino acid R14 is underlined. Examination of the PKA motif recognition sequence determined the requirement of two arginine (R) residues (red arrow) just downstream from the serine to be phosphorylated, in contrast to the CaMKIID motif sequence logos of the consensus PKA or CaMKIID recognition sequences derived by PhosphoSite [36,37].

To investigate why the PLN-R14del/SERCA2a interaction cannot be modulated by phosphorylation, we examined the actual levels of PLN-R14del phosphorylation at the Ser-16 site, which is the residue phosphorylated by PKA [1]. In vitro phosphorylation by the PKA treatment of GST-PLN-R14del recombinant protein unveiled that no Ser-16 phosphorylation could be achieved (Figure 4a). This finding was further confirmed by ISO treatment in transfected HEK 293 cells (Figure 4b).

In search for the explanation as to how the R14del mutation could be impacting Ser-16 phosphorylation, we examined the PLN amino acid sequence surrounding the R14 residue. Initially, we aligned PLN sequences from difference species and we determined significant conservation, especially around R14 and the Ser-16 sites, therefore suggesting their critical involvement in PLN function (Figure 4c). We next analyzed the PKA motif consensus sequence, which demonstrated the essential requirement of two arginine (R) residues just downstream from the serine to be phosphorylated (Figure 4c). As one of these residues is removed by the PLN-R14del mutation, its absence results in disruption of the PKA consensus recognition sequence and, thus, failure of PLN-R14del phosphorylation at the Ser-16 site. On the other hand, analysis of the Ca^2+^/calmodulin dependent protein kinase II delta (CaMKIID) motif revealed the requirement of only one R residue (Figure 4c), and, therefore, R13 alone likely suffices in the PLN-R14del mutant protein, despite the lack of R14. This suggests that CaMKII-mediated phosphorylation at the Thr-17 site will not be affected in PLN-R14del, in agreement with previous in vitro findings [35].

### 2.4. PLN-R14del Exhibits Phsysiological Subcellular Distribution and Co-Localizes with SERCA2a

**Figure 5:**
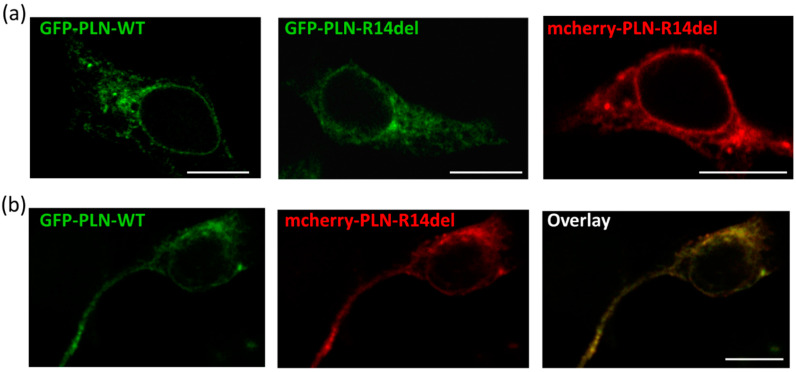
PLN-R14del exhibits normal subcellular localization. (**a**) In HEK 293-transfected cells, GFP-PLN-R14del shows a similar localization pattern to GFP-PLN-WT. An analogous subcellular distribution is also observed for the mcherry-PLN-R14del construct. (**b**) Upon co-transfection, co-localization of the GFP-PLN-WT and mcherry-PLN-R14del proteins is observed. Scale bar: 10 μm.

To determine whether the presence of the R14del mutation had an effect beyond the significant aberrations in protein interactions, we proceeded to assess whether PLN-R14del exhibited alterations in its subcellular distribution in our cell culture system. We initially examined the subcellular localization of PLN-R14del in HEK 293 cells. No major alterations were observed in the localization pattern of either GFP-PLN-R14del or mcherry-PLN-R14del constructs, which, in agreement with previous findings [5], exhibited ER and perinuclear localization, similar to GFP- PLN-WT (Figure 5a). Moreover, upon co-transfection of PLN-WT and PLN-R14del proteins, extensive co-localization was observed between GFP-PLN-WT and mcherry-PLN-R14del, thus confirming the physiological subcellular distribution of PLN-R14del (Figure 5b).

**Figure 6:**
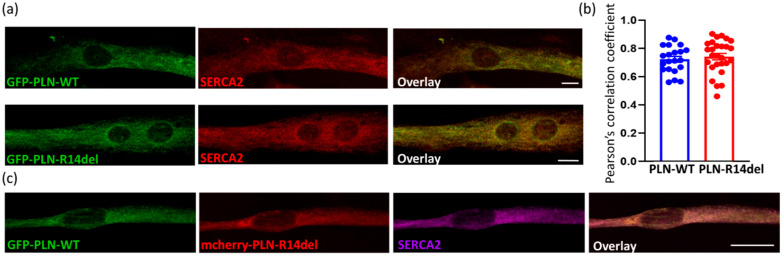
PLN-R14del co-localizes with SERCA2a in cardiac cells. (**a**) In differentiated H9c2 cells, GFP-PLN-R14del co-localizes with endogenous SERCA2a protein. (**b**) Calculation of Pearson’s correlation coefficient determined a similar co-localization to SERCA2a for GFP-PLN-WT and GFP-PLN-R14del. Data are the mean ±SE; *n*=21–27 cells from each group. (**c**) mcherry-PLN-R14del co-localizes with GFP-PLN-WT and SERCA2a. Scale bar: 10 μm.

In parallel, we evaluated the localization of PLN-R14del in the cardiac cell line H9c2. Following transfections, cells were differentiated for 7 days prior to assessing the co-localization of GFP-PLN-R14del to the endogenous SERCA2a expressed by these cells. By immunofluorescence analysis, we determined extensive co-localization between PLN-R14del and SERCA2a, occurring at similar levels to PLN-WT (Figure 6a,b). Moreover, when GFP-PLN-WT was co-transfected with mcherry-PLN-R14del, both proteins were shown to co-localize to SERCA2a (Figure 6c).

### 2.5. In Silico Prediction of Alterations in PLN-R14del Structure

**Figure 7:**
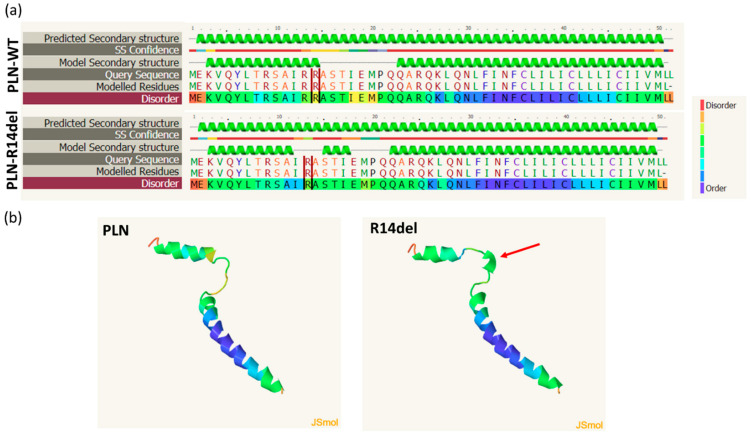
PLN-R14del structure alterations. (**a**) Predicted secondary structure of PLN-WT or PLN-R14del human proteins reveals changes in the mutant protein. Residue 14 is indicated by red lines in PLN-WT but is deleted in the PLN-R14del sequence. Green helices represent α-helices while grey lines indicate coils. (**b**) Structural views of PLN-WT and PLN-R14del proteins depicting the impact of the extra α-helix (shown by red arrow) in the structure of PLN-R14del.

As PLN-R14del exhibits a similar localization pattern to PLN-WT, we proceeded to investigate whether the mutation may cause structural alterations that could affect its physiological functions. In silico prediction analysis was performed using the Phyre2 web portal for protein modeling and prediction analysis [38]. For this, the human PLN-WT or PLN-R14del protein sequences were entered on Phyre2 [39], generating a structural model based on templates already available in the protein data bank (PDB) database. For both PLN-WT and PLN-R14del sequences, the predicted model was based on the NMR structure of unphosphorylated human PLN pentamer (PDB: c1zllE). Detailed bioinformatical analysis using the Phyre Investigator tool resulted in the generation of secondary structure prediction models. When compared to PLN-WT, considerable changes were observed in the predicted secondary structure of PLN-R14del. In particular, deletion of residue 14 appears to disrupt the coil domain of the protein by introducing an additional α-helix just downstream from the deletion and around amino acid residues STI of the mutant protein (Figure 7a). Moreover, moderate disorder prediction changes were observed (21% disorder for PLN-WT protein vs. 14% for PLN-R14del), occurring specifically around the region of the mutation. This finding suggests possible defects in PLN-R14del flexibility, which could contribute to overall changes in the protein structure. Indeed, this becomes evident by direct comparison of the structural views of PLN-WT and PLN-R14del proteins (Figure 7b). These structural changes would be expected to impact PLN-R14del functional properties, such as its protein interactions, and could, therefore, underlie aberrations implicated in disease pathophysiology.

## 3. Discussion

The human *PLN* mutation R14del has been reported in an increasing number of patients around the world, which present with clinical features of arrhythmogenic cardiomyopathy (ACM) and DCM [3,10]. Although the genetic etiology of disease is now well defined, the molecular mechanisms underlying PLN-R14del pathogenesis remain unclear. We focused on the assessment of the immediate first steps in the pathogenetic process at the structural, functional, and topological levels. We present evidence to support that PLN-R14del exhibits impaired associations to SERCA2a and HAX-1, two key PLN-interacting partners that regulate Ca^2+^ homeostasis. Moreover, as the PLN-R14del protein does not become phosphorylated at the Ser-16 residue, PLN-R14del remains bound to SERCA2a even upon β-adrenergic stimulation or upon increased demand of cardiac output to the periphery during exercise or stress conditions. These findings reveal that PLN-R14del acts as a nonreversible inhibitor of SERCA2a, causing impaired SR Ca^2+^ cycling and depressed cardiac function that are not relieved even upon β-adrenergic stimulation.

Emerging evidence from PLN-R14del mouse models, as well as human-derived cardiomyocytes, has established aberrant Ca^2+^ cycling as an underlying pathology of the PLN-R14del mutation [13,16,18]. In the recently reported humanized PLN-R14del mouse model, detailed analysis of contractile parameters revealed considerable SR Ca^2+^ defects, including depressed Ca^2+^ kinetics and contractile parameters, as well as elevated diastolic Ca^2+^ levels due to the super-inhibition of SERCA2a activity. This was found to trigger increased Ca^2+^-leaks and Ca^2+^ sparks, which were associated with increased CaMKII activity. Consequent aberrations included the prolongation of action potential duration, increased spontaneous aftercontractions, and increased propensity to arrhythmias [16]. In particular, while infrequent premature ventricular contractions were observed under basal conditions, PLN-R14del mice exhibited stress-induced atrioventricular conduction impairment, delayed ventricular activation, prolonged repolarization, and ventricular tachyarrhythmias that originated from the right ventricle [16]. Importantly, most of these electrocardiographic alterations were similar to those seen in PLN-R14del patients [16]. The measurement of SERCA2a function, following PLN-R14del overexpression in HEK 293 cells, determined that PLN-R14del exerts super-inhibitory effects on SERCA2a activity [5]. Our findings are the first to provide molecular insights into this aberration, suggesting that this super-inhibitory effect is due to increased PLN-R14del/SERCA2a binding. Furthermore, we show that disruption of the PKA sequence motif due to deletion of the R14 residue leads to the inability of the mutant protein to become phosphorylated at the Ser-16 site. The importance of the PKA consensus motif as a prerequisite for PKA-dependent phosphorylation has been shown in additional cardiac proteins [40]. Consequently, the PLN-R14del/SERCA2a association persists irrespective of phosphorylation signals, leading to the continuous suppression of SERCA2a activity. Indeed, and in agreement with our findings, SERCA2a inhibition and contractile parameters in PLN-R14del mouse hearts are not fully relieved upon phosphorylation [5,16]. Another layer contributing to the inhibition of SERCA2a activity may be mediated through HAX-1, a protein that has been shown to increase the PLN inhibitory action on SERCA2a [26,41]. Thus, the inhibitory effects of mutant PLN are mediated by both its increased interaction with SERCA2a as well as HAX-1, resulting in an overall super-inhibition of the Ca^2+^-affinity of the SR Ca^2+^-transport system. Importantly, as the super-inhibitory action of PLN-R14del is not relieved even upon β-adrenergic stimulation, this results in chronic suppression of SERCA2a activity, which may lead to the observed cardiac remodeling in PLN-R14del patients and mouse models [5,9,11,42].

In addition to PLN, the SERCA2a function is regulated by HRC. HRC binds directly to SERCA2a and acts as a dual modulator of Ca^2+^ cycling, being implicated in the regulation of both SR Ca^2+^ uptake and SR Ca^2+^ release [27,43,44]. In the presence of PLN-R14del, the observed enhanced HRC/SERCA2a interaction may result in inhibition of the maximal velocity of SERCA2a [31,43] and further contribute to the overall inhibition of SR Ca^2+^-uptake. This preferential binding of HRC to SERCA2a in the presence of PLN-R14del will consequently result in less HRC associated with the SR Ca^2+^ release complex. This, in turn, could lead to the aberrant regulation of SR Ca^2+^-cycling [31,43,45] and promote increased Ca^2+^ leak and arrhythmogenic propensity, as have been observed in the humanized PLN-R14del mouse [16]. Interestingly, in addition to PLN-R14del, a human variant of HRC (HRC-Ser96Ala) as well as another PLN mutation (PLN-R25C) have both been shown to act as super-inhibitors of SERCA2a, an effect that is also mediated through their enhanced interaction to SERCA2a [46,47]. Importantly, in both cases, this causes SR Ca^2+^ cycling defects that ultimately lead to increased SR Ca^2+^ sparks and arrhythmias [46,47]. Collectively, this suggests that the enhanced association to SERCA2a and super-inhibition of its activity represents a common pathological mechanism underlying impaired SR Ca^2+^ cycling, depressed cardiac function, and arrhythmogenesis.

At the cellular level, PLN-R14del was previously proposed to exhibit some alterations in its subcellular distribution [18,48]. This was based on studies in either patient-derived iPSC-CMs that presented with polarized distribution of PLN-R14del at one side of the cytoplasm, or cardiac tissue from a PLN-R14del mouse model that expressed the mutant protein in the PLN-null background [18,48]. In this mouse model, in the absence of endogenous PLN-WT, the mouse PLN-R14del was mis-localized to the plasma membrane where it interacted with the sarcolemmal Na/K-ATPase (NKA) [48]. However, recent immunofluorescence studies in cardiomyocytes from heterozygous mice of the humanized PLN-R14del model revealed a physiological distribution for the PLN-R14del and its co-localization with SERCA2a in both left-ventricular and right-ventricular cardiomyocytes [16]. In agreement with this, we also observe a physiological subcellular distribution of PLN-R14del in both HEK 293 and H9c2 cardiac cells, where it co-localizes with both PLN-WT and SERCA2a. The use of fluorescently tagged proteins adds considerable strength to our experimental approach as it overcomes the previously encountered challenge of using PLN antibodies for localization purposes [5]. Our study, therefore, provides clear evidence to demonstrate the subcellular localization pattern of the PLN-R14del mutant protein itself in differentiated cardiomyoblast cells.

The mechanism contributing to the super-inhibitory effect of PLN-R14del on SERCA2a may be associated with conformational changes of the mutant protein. The high conservation of the residue R14 among species and its localization within a structural region of PLN’s cytosolic domain highlight its significance in the PLN structure. Indeed, upon deletion of R14, our in silico predictions indicate considerable alterations in the PLN-R14del structure. In particular, R14del appears to disrupt the mutant protein’s coil domain, an essential structure that forms a hinge that connects the two α-helical stretches of the protein [49,50]. The presence of the coil domain is believed to provide flexibility that enables conformational changes associated with PLN phosphorylation and association with SERCA2a [50]. Previous analysis by nuclear magnetic resonance on PLN-R14del reconstituted with SERCA2a-containing lipid membranes determined slight perturbations in the helical structure of PLN-R14del’s cytoplasmic domain, showing reduced affinity for the phospholipid bilayer surface [51]. These structural changes were associated with perturbations in PLN-R14del conformational dynamics and impaired SERCA2a regulation [52]. Overall, these findings demonstrate the critical role of maintaining the PLN structure toward fine-tuning SERCA2a function.

**Figure 8:**
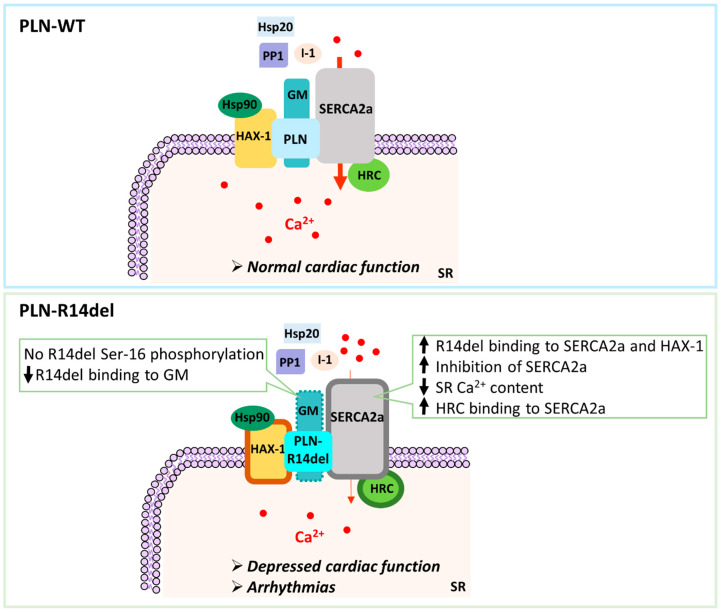
Schematic representation of the molecular defects associated with the PLN-R14del mutation. Changes in protein binding are depicted as thick outlines (increased interaction) or dashed lines (decreased interaction). Resultant alterations in SERCA2a activity are indicated by modifications in the red arrow thickness (thin arrow represents reduced activity), while impaired SR Ca^2+^ cycling processes are illustrated by differences in the amount of Ca^2+^ (red circles).

Based on the findings presented in this study, and taking into consideration the described pathophysiological features of human patients and PLN-R14del mouse models [5,10,16,18], we propose the following hypothesis on the molecular defects that impair SR Ca^2+^ homeostasis in PLN-R14del hearts (Figure 8): PLN-R14del exhibits enhanced association to SERCA2a, possibly due to structural changes of the mutant protein. As a result, SERCA2a activity is inhibited, causing decreased SR Ca^2+^ uptake and depressed contractility. Importantly, the increased PLN-R14del/SERCA2a association is maintained even upon PKA-stimulation due to the lack of PLN-R14del phosphorylation at the Ser-16 site. As a compensatory mechanism, binding to the phosphatase targeting subunit GM is reduced. At the same time, the interaction of HRC to SERCA2a is enhanced by PLN-R14del, leading to the additional inhibition of SR Ca^2+^-transport and increases in diastolic Ca^2+^ levels. Overall, this cascade of molecular and sub-cellular events is consistent with the increased aftercontractions and prolongation of the action potential duration that have been described in the PLN-R14del hearts. Although the use of heterologous expression systems such as HEK 293 or H9c2 cells as well as mouse models present with certain limitations, when aiming to unravel a human disease [53,54,55], they can and have offered valuable insights on pathogenetic mechanisms, which serve as the basis for subsequent, finely targeted studies in precious patient tissues.

Collectively, our study provides the first evidence on the direct implications of the PLN-R14del mutation on its immediate interactions, and the impairment of those that may lead to a series of molecular aberrations associated with aberrant SR Ca^2+^ handling and ultimately arrhythmogenesis. Future studies designed toward relieving SERCA2a super-inhibition could provide promising therapeutic strategies for PLN-R14del patients.

## 4. Materials and Methods

### 4.1. Generation of Recombinant Proteins

The generation of the GST-PLN-WT expression construct has been previously described [25]. For the generation of the GST-PLN-R14del construct, the PLN-R14del/pBluescript construct [5] was used as a template for PCR amplification and the PCR product was subsequently cloned in the pGEX 5x-1 vector (Amersham Biosciences, Uppsala, Sweden), as previously described [25]. The authenticity of all constructs was confirmed by sequence analysis (Macrogen Europe, Amsterdam, The Netherlands). Protein expression was performed as previously described [25] and recombinant proteins were purified on Protino Glutathione Agarose 4B (Macherey Nagel, Dueren, Germany) according to the manufacturer’s instructions.

### 4.2. Pull-Down Assays

Pull-down assays were performed as previously described [25,56]. Briefly, wild-type mouse cardiac homogenates were prepared in 10 mM NaPO_4_ (pH 7.2), 2 mM EDTA, 10 mM NaN_3_, 120 mM NaCl, and 1% NP-40, supplemented with protease inhibitors (Sigma-Aldrich, Munich, Germany). Equivalent amounts of recombinant GST-PLN-WT and GST-PLN-R14del recombinant proteins were mixed with 0.5 mg of mouse cardiac homogenates at 4 °C for 16 h. The beads were washed with 10 mM NaPO_4_ (pH 7.2), 10 mM NaN_3_, 120 mM NaCl, and 0.1% (*v/v*) Tween-20 and were subsequently analyzed by Western blot with the following primary antibodies: SERCA2, Hsp90 (Cell Signaling Technology, Leiden, The Netherlands), HAX-1 (BD Biosciences, Erembodegem, Belgium), PP1, GM (Santa Cruz Biotechnology, Heidelberg, Germany), Hsp20, I-1 (AbCam, Cambridge, UK), HRC (Sigma-Aldrich, Munich, Germany), and peroxidase-conjugated goat anti-rabbit (GE Healthcare Life Sciences, Buckinghamshire, UK) or anti-mouse (Sigma-Aldrich, Munich, Germany) secondary antibodies. Immunoreactive bands were detected using Pierce ECL Plus reagents (ThermoFisher Scientific, Waltham, MA, USA). Protein quantification was performed using ImageJ [57].

### 4.3. Immunoprecipitations in Mouse Hearts

Hearts from PLN-WT or PLN-R14del transgenic mice were homogenized with 1x cell lysis buffer (Cell Signaling Technology, Leiden, The Netherlands), supplemented with protease inhibitor cocktail (Millipore Sigma) and phosphatase inhibitor cocktail sets I and II (Calbiochem, Merck, Darmstadt, Germany), and were centrifuged at 10,000 rpm for 30 min at 4 °C. Protein homogenates were diluted to 1 g/L (1 mL of final volume) and were incubated with the SERCA2 antibody (ThermoFisher Scientific, Waltham, MA, USA) at 4 °C for 16 h. An amount of 100 μL of protein G PLUS agarose beads (Santa Cruz Biotechnology, Heidelberg, Germany) were then added into the mixture and the samples were incubated for an additional 5 h. Agarose beads were then sedimented and were washed 6 times with cell lysis buffer. Beads-bound proteins were dissociated in 2 × SDS at room temperature for 30 min with vortexing at 5 min intervals. Samples were analyzed by Western blot using PLN (ThermoFisher Scientific, Waltham, MA, USA) and HAX-1 (BD Biosciences, Erembodegem, Belgium) antibodies.

### 4.4. Cell Culture, Transfections, and Immunofluorescence Studies

HEK 293 cells (ECACC, Salisbury, UK) were maintained in Dulbecco’s modified Eagle’s medium (DMEM) supplemented with 10% fetal bovine serum (FBS) (ThermoFisher Scientific, Waltham, MA, USA), as previously described [25,47]. The GFP-PLN-WT construct has been previously described [25], while for the generation of the GFP-PLN-R14del construct, the PLN-R14del/pBluescript plasmid [5] was used as a template for PCR amplification. The PCR product was subsequently cloned in the pEGFP vector (BD Biosciences Clontech, Erembodegem, Belgium), as previously described [25]. In the case of the mcherry-PLN-R14del construct, the mcherry fluorescent tag was excised by BsrGI/NdeI digestion from an mcherry expressing construct (kind gift from Dr Costis Papanayotou). In parallel, BsrGI/NdeI digestion of the GST-PLN-R14del plasmid allowed replacement of the GFP moiety with the mcherry fluorescent tag. The authenticity of all constructs was confirmed by sequence analysis (Macrogen Europe, Amsterdam, The Netherlands). Transient transfections in HEK 293 cells were performed using Lipofectamine™ 2000 (ThermoFisher Scientific, Waltham, MA, USA), according to the manufacturer’s instructions.

H9c2, a rat heart myoblast cell line (ECACC), was maintained in DMEM supplemented with 10% FBS (ThermoFisher Scientific). Transient transfections in H9c2 cells were performed using Viromer Red (Lipocalyx GmbH, Halle, Germany), according to the manufacturer’s instructions. Cell differentiation and myotube formation were induced by switching to differentiation media (DMEM supplemented with 1% FBS and 10 nM retinoic acid).

Immunofluorescence studies were performed after 7 days of differentiation, as previously described [25,58]. In brief, cells were fixed for 20 min at 25 °C with ice-cold methanol, washed three times with phosphate-buffered saline (1× PBS), and permeabilized for 30 min at 25 °C in PBS containing 0.1% Triton X-100. The cells were then washed in PBS prior to incubation with blocking buffer (1× PBS, 1 mg/mL of BSA, and 10 mM NaN_3_) for 1 h at 25 °C. The SERCA2 antibody (Cell Signaling Technology, Leiden, The Netherlands) was diluted in blocking buffer and applied to the cells for 1 h at 25 °C. Following three washes with PBS, cells were counterstained for 1 h at 25 °C with the Alexa Fluor anti-rabbit 633 (Invitrogen, ThermoFisher Scientific, Waltham, MA, USA) secondary antibody diluted in blocking buffer. After three washes with PBS, samples were mounted with Fluoroshield mounting medium with DAPI (Abcam, Cambridge, UK) and analyzed on a Leica confocal laser scanning microscope (Leica TCS SP5 on a DMI6000 Inverted Microscope, with the acquisition software program LAS-AF). Co-localization analysis was performed using the Colocalization Threshold plugin of ImageJ [57].

### 4.5. Immunoprecipitations in Transiently Transfected HEK 293 Cells

Immunoprecipitation experiments in transiently transfected HEK 293 cells were performed as previously described [30,47]. Briefly, forty-eight hours after transfection, cells were lysed in 50 mM Tris HCl, 150 mM NaCl, and 1% NP40 supplemented with protease inhibitors. Pre-cleared protein extracts were incubated overnight on a rotary wheel at 4 °C with the GFP antibody (Sigma-Aldrich, Munich, Germany, Erembodegem, Belgium) and protein-A/G agarose beads (Santa Cruz Biotechnology, Heidelberg, Germany). Immunoprecipitates were collected by a 5 min spin at 2000 rpm, washed three times in PBS, and analyzed by Western blot analysis using SERCA2 (Cell Signaling Technology, Leiden, The Netherlands) or GFP (Sigma-Aldrich, Munich, Germany) antibodies.

### 4.6. Protein Phosphorylation

In order to evaluate the effect of phosphorylation on SERCA2a binding, transfected HEK 293 cells were phosphorylated by treatment with 10 μM isoproterenol for 30 min at 37 °C [59]. Subsequently, cells were lysed and immunoprecipitation assays were performed, as described above. PLN phosphorylation was evaluated using the phospho-Ser16 PLN antibody (AbCam, Cambridge, UK).

In parallel, in vitro protein phosphorylation of GST-PLN or GST-PLN-R14del recombinant proteins was performed using the cAMP-dependent Protein Kinase (PKA) catalytic subunit (New England Biolabs, Ipswich, Massachusetts, USA), as previously described [30,47]. Briefly, proteins were incubated with 1X PKA Reaction Buffer (50 mM Tris-HCl pH 7.5, 10 mM MgCl_2_), supplemented with 200 μM ATP (Millipore) and 1250 units of PKA catalytic subunit. Samples were incubated at 30 °C for 1 h and were subsequently used in pull-down assays, as described above.

## Data Availability

Not applicable.

## Abbreviations

PLN	Phospholamban
SERCA2a	Sarcoplasmic reticulum (SR) calcium ATPase
HAX-1	HS-1-associated protein X-1
GM	Muscle-specific glycogen-targeting subunit of PP1
PP1	Protein phosphatase 1
Hsp20	Heat shock protein 20
I-1	Inhibitor-1
Hsp90	Heat shock protein 90
HRC	Histidine-rich calcium binding protein
PKA	cAMP-dependent protein kinase
ISO	Isoproterenol
DCM	Dilated cardiomyopathy
ACM	Arrhythmogenic cardiomyopathy
